# Future Directions and Priorities for Cellular Therapy in Sarcoma: A Report from the Strategic Advances in Sarcoma Science Cell Therapy Breakout

**DOI:** 10.3390/cancers17183068

**Published:** 2025-09-19

**Authors:** Jacqueline Oliva-Ramirez, David Milewski, Lauren Banks, Kelly M. Bailey, Everett J. Moding, Jessica Lake, Alice Chen, Jessica D. Daley, Erin E. Resch, Rosandra N. Kaplan, Brian H. Ladle, Lindy Zhang, Margaret M. Chou, Rosa Nguyen, Urania Dagalakis, Nourhane Al Akoum, Poul H Sorensen, Jonathan A. Fletcher, Ronald DeMatteo, Nicolas J. Llosa, Seth M. Pollack

**Affiliations:** 1 Department of Translational Molecular Pathology, MD Anderson Cancer Center, Houston, TX 77030, USA; 2 Genetics Branch, Center for Cancer Research, National Cancer Institute, National Institutes of Health, Bethesda, MD 20892, USA; david.milewski@nih.gov; 3 Sarcoma Medical Oncology Service, Memorial Sloan Kettering Cancer, New York, NY 10065, USA; 4 Division of Pediatric Hematology-Oncology, University of Pittsburgh School of Medicine, Pittsburgh, PA 15215, USAdaleyjd@upmc.edu (J.D.D.); 5 Department of Radiation Oncology, Stanford University School of Medicine, Stanford, CA 94305, USA; emoding@stanford.edu; 6 Pediatric Oncology Branch, Center for Cancer Research, National Cancer Institute, National Institutes of Health, Bethesda, MD 20892, USA; jessica.lake@nih.gov (J.L.);; 7 Division of Cancer Treatment and Diagnosis, National Cancer Institute, National Institutes of Health, Bethesda, MD 20892, USA; 8 Division of Pediatric Oncology, Department of Oncology, The Sidney Kimmel Comprehensive Cancer Center, Johns Hopkins University School of Medicine, Baltimore, MD 21287, USA; bladle@jhmi.edu (B.H.L.);; 9 Department of Pathology and Laboratory Medicine, Children’s Hospital of Philadelphia and the Perelman School of Medicine, Philadelphia, PA 19104, USA; mmc@pennmedicine.upenn.edu; 10 Division of Hematology and Oncology, Northwestern University Feinberg School of Medicine, Chicago, IL 60611, USA; nourhane.alakoum@northwestern.edu; 11 Department of Pathology and Laboratory, University of British Columbia, Vancouver, BC V5Z 1L3, Canada; 12 Department of Pathology, Brigham and Women’s Hospital, Boston, MA 02115, USA; 13 Department of Surgery, Memorial Sloan Kettering Cancer Center, New York, NY 10065, USA

**Keywords:** sarcoma, immunotherapy, TCR, chimeric antigen receptor, adoptive cellular therapies (ACTs)

## Abstract

Background: In September of 2024, the 2nd annual meeting of the Strategic Advances in Sarcoma Science (SASS) convened at the National Institutes of Health. This gathering of national sarcoma experts focused on preclinical studies, clinical trials, opportunities, challenges, and future directions in sarcoma biology and clinical care with a focus on immunotherapy. The Immunology in Sarcoma breakout group conducted a dedicated discussion focused on the current and future implementation of adoptive cellular therapies (ACTs) in sarcomas. The current manuscript summarizes these discussions and provides a comprehensive resource for researchers and clinicians. Results: Adoptive cell therapy (ACT) has shown encouraging results in sarcomas with afami-cel achieving durable responses in synovial sarcoma and early TCR-T trials against NY-ESO-1 and MAGE-A4 demonstrating meaningful response rates. Building on these outcomes will require discovering new targets, selecting optimal cell types, refining conditioning regimens, combining with alternative treatment strategies such as TKIs, and leveraging predictive biomarkers informed by a deeper understanding of the tumor microenvironment. Conclusions: Sarcomas are promising targets for adoptive cell therapy (ACT), as shown by afami-cel’s success in synovial sarcoma, but broader impact requires new target discovery, optimal cell selection, improved conditioning, combination treatments, deeper tumor microenvironment understanding, and predictive biomarkers to achieve more durable responses for more patients.

## Simple Summary

Sarcoma experts at the Strategic Advances in Sarcoma Science met to discuss recent progress in preclinical models, clinical trials, and translational opportunities, while addressing key challenges and future perspectives related to adoptive cellular therapy. This manuscript highlights those discussions, offering not only a comprehensive resource for researchers and clinicians in the field but also a deeper understanding of the implementation of adoptive cellular therapies (ACTs) in the treatment regimen.

## 1. Introduction

Sarcomas, broadly categorized as soft tissue and bone sarcomas, represent approximately one hundred distinct pathologic entities and arise from the connective tissues [1,2]. Sarcomas are considered rare malignancies, comprising ~20% of pediatric and ~1% of adult solid tumors [3]. Greater than 50 sarcomas meet the criteria of an ‘ultra-rare’ cancer, which is a designation defined by an incidence of ≤1 case per 1,000,000 people [4]. The rarity of sarcomas presents a clear challenge for the prospective testing of novel treatments in clinical trials and ensuring access to sarcoma specialty care in underserved areas [5,6,7,8]. Although primary sarcomas can often be cured with local therapies like surgery and radiotherapy, many sarcomas have high rates of metastatic disease [9,10]. In patients with metastatic sarcomas, there are limited effective treatment options, and almost all patients progress after conventional chemotherapy [11,12]. In the past decade, immunotherapies such as checkpoint inhibitors have shown promise in some patients, such as those diagnosed with metastatic alveolar soft part sarcoma, undifferentiated pleomorphic sarcoma (UPS) and liposarcoma (LPS); however, the majority of sarcomas demonstrate no meaningful response to first-generation checkpoint inhibition targeting PD-1, PD-L1, and CTLA-4 [13,14,15].

**Figure 1:**
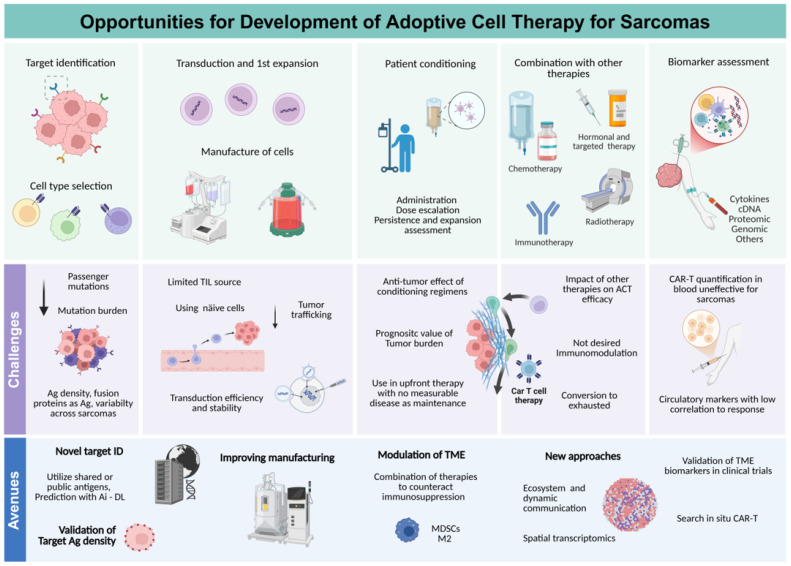
Adoptive cell therapy in sarcoma: modalities, targets, trials and challenges. Created in BioRender by Oliva-Ramírez, J. (2025) www.biorender.com (accessed on 1 September 2025).

Sarcomas present several biology-based treatment challenges [16]. Sarcomas are driven by diverse molecular alterations ranging from characteristic translocations to complex karyotypes associated with chromosomal instability [17,18,19]. Although oncogenic drivers have been successfully targeted in a subset of sarcomas, targeted therapies do not exist for the majority of sarcoma subtypes [20,21]. Furthermore, sarcomas demonstrate inter- and intra-tumoral heterogeneity [22,23,24] with tumor cell plasticity contributing to therapy resistance [25,26,27]. The tumor microenvironment (TME) plays a critical role in sarcoma progression and response to therapy [28,29], and the microenvironment may vary between the primary tumor and metastatic disease in different organs [30]. A deeper understanding of sarcoma immunobiology is essential for rational therapeutic advances. Novel single agents or combination strategies to target vulnerabilities of individual sarcoma s subtypes are greatly needed (Figure 1).

## 2. Results

### 2.1. Adoptive Cell Therapy

Adoptive cell therapy (ACT) has revolutionized cancer treatment, eliciting durable remissions and even cures in some patients with advanced disease [31,32,33,34]. In ACT, immune cells (most commonly autologous CD8^+^ T cells) are genetically modified in vitro to express receptors that enhance the recognition and killing of cancer cells; then, they are re-infused into patients. T cell ACTs can be directed against surface antigens expressed by tumor cells using chimeric antigen receptors (CARs), intracellular antigens presented by the major histocompatibility complex (MHC) using genetically engineered T cell receptors (TCRs), or peptide-centric (PC) CARs [35,36,37]. To date, the Food and Drug Administration (FDA) has approved seven CAR-T cell therapies, all for the treatment of hematological malignancies [34]. However, recent clinical results have demonstrated the tremendous potential of ACT in sarcomas. The SPEARHEAD-1 clinical trial of afamitresgene autoleucel (afami-cel), an engineered TCR-T cell therapy against MAGE-A4, demonstrated a 39% response rate in heavily pre-treated patients with synovial sarcomas [38], leading to the first FDA approval of an engineered T-cell therapy in solid tumors. Similarly impressive responses were recently reported for the IGNYTE-ESO trial in synovial sarcoma and myxoid/round cell liposarcomas treated with letetresgene autoleucel (lete-cel), which is a TCR-T therapy targeting NY-ESO-1 [39]. Multiple additional CAR-T cell and TCR-T cell therapies are currently under preclinical and clinical investigation for various bone and soft tissue sarcomas, targeting antigens that include NY-ESO-1, GD2, interleukin-1 receptor accessory protein (IL1RAP), HER2, and B7-H3 [40,41,42,43]. Due to the ability of natural killer (NK) cells to mediate killing in an antigen-independent manner, many NK cell ACTs are also currently in clinical development [44,45,46,47]. Sarcomas are among the cancers most sensitive to NK-mediated cytolysis, and NK cells have been implicated in the immune surveillance of sarcoma during their initiation, progression, and metastasis [44]. As a result, CAR-NK cells are being avidly explored for multiple sarcoma subtypes, including osteosarcoma, Ewing sarcoma, and rhabdomyosarcoma [44,45].

Multiple challenges must be overcome to realize the full potential of ACT in the management of sarcoma. One critical objective is the identification of novel target antigens. The mesenchymal origin of sarcomas may make them particularly vulnerable to cellular therapies due to expression of antigens that are not expressed in most normal cells [48]. Prior studies have shown that many sarcomas express cancer testis antigens that are typically not found in somatic normal tissues [43,49]. Furthermore, many sarcomas are driven by translocations that lead to the expression of fusion proteins representing tumor-specific targets for cellular therapies [50]. Despite the great potential of T cell-based ACT, efficacy is limited by its dependence on target antigen expression and MHC expression in the case of engineered T cells, which can lead to primary or acquired resistance [31,32,33]. CAR-NK therapy may overcome this limitation by targeting antigen-expressing cells while retaining innate cytotoxicity in the event of antigen loss [47]. The immunosuppressive TME creates formidable hurdles in solid tumors, necessitating the development of novel approaches to promote intra-tumoral recruitment and penetration, persistence, and effector function while preventing the exhaustion of engineered T and NK cells and promoting memory formation for lasting anti-tumor immunity [31,32,33,51,52,53,54,55]. Furthermore, increased focus should be directed toward methods to counteract the immunosuppressive, pro-tumorigenic functions of immune lineages that are abundant in the sarcoma TME, such as regulatory T cells, M2 macrophages and myeloid-derived suppressor cells (MDSCs) [51,52,56,57,58]. Continued progress toward addressing these various challenges raises optimism for the development and preclinical testing of novel immunotherapeutics that aim to improve outcomes for patients with aggressive sarcomas that are refractory to conventional therapies.

### 2.2. Target Identification

The underlying assumption in immune targeting of cancers using ACT with immune effector cells is that tumor cells aberrantly express proteins and carry passenger or driver mutations that exist in cancer cells but have little to no expression in normal healthy cells. These changes are thought to generate two classes of targetable antigens: tumor-associated antigens (TAAs), including tumor differentiation antigens (TDAs) that might be expressed in normal tissues but are significantly overexpressed in tumor tissue, and tumor-specific antigens (TSAs), which are expressed only in tumor tissue [35]. Tumor antigens can be targeted in two ways:

1. As membrane-associated proteins;
2. As part of a peptide-human leukocyte antigen (pHLA) complex after antigens undergo proteasomal cleavage and are loaded into HLA molecules for trafficking to and presentation on the cell surface. ACT therapies broadly make use of one of two mechanisms of antigen recognition: antibody-based approaches relying on high affinity binding between immune receptors and intact surface proteins or TCR-based approaches using either TCRs or TCR-mimics relying on a TCR recognition pattern of pHLA complexes [59]. This discussion will focus on the latter and the identification of TCR targets in sarcomas.

While some TAAs and TSAs are unique to a specific tumor cell type and acquired over time through passenger mutations, targeting these “private” or individualized antigens can be costly and cumbersome, leading to a therapeutic pipeline that is not easily translated into the clinic. Additionally, most sarcomas have few of these passenger mutations and an overall low mutational burden [19]. Therefore, another approach to target identification in sarcomas is to first identify shared, or “public,” tumor antigens. Such examples are common driver mutations, fusion proteins, or cancer germline antigens transcriptionally upregulated by oncogenic drivers such as NY-ESO-1 or MAGE-A4 [60,61,62,63,64]. If derived from TAAs or TDAs, expression on normal cells needs to be limited to avoid off tumor multiorgan toxicity. A recent plasma enrichment strategy to identify candidate surface proteins for immunotherapy in Ewing sarcoma may be generally applicable across other sarcoma types [65]. Ideally, these antigens should be essential to the intrinsic biology of the tumor cell and its tumorigenesis such that they cannot be lost as an immune escape mechanism [59,65,66].

While immunohistochemistry or next-generation sequencing (NGS) data can help identify candidate proteins, and in silico prediction algorithms such as NET-MHC can predict if epitopes derived from them can bind common HLA molecules, there is an increasing appreciation for the need to empirically verify the presence of tumor antigen pHLA on the surface of tumor cells using immunopeptidomics [64,67]. The gold standard on this front is HLA-immunoprecipitation with the liquid chromatography/mass spectrometry-based identification of HLA-bound tumor antigens sourced from tumor tissue or cells combined with tumor NGS data [68,69]. Immunopeptidomics interrogates the following required elements of antigen presentation not captured in IHC, NGS, or in silico results alone:

1. Expression of HLA molecules by tumor cells;
2. Proteasomal cleavage of the tumor protein such that an epitope capable of binding the HLA of interest is generated; and
3. Adequate antigen-presenting machinery to load the cleaved epitope into HLA molecules and traffic pHLA to the cell surface [64,70]. Yet another important component to take into consideration is the proteasome invoked, as there are differential cleavage patterns between the classical and immune proteasomes [71]. For example, an immunogenic tumor antigen may only be processed and presented when the components of the immune proteasome are upregulated in the presence of IFNγ [72]. Supporting this assertion, downregulation of the immune proteasome has been demonstrated as a mechanism of “antigen loss” whereby the target is not presented on the surface as a functional resistance mechanism in acute myeloid leukemia (AML) after WT-1 targeted TCR therapy [73].

Understanding optimal targets cannot be divorced from immune effector receptor development and functional avidity, which is determined by both immune receptor affinity and antigen density. TCR affinity for antigens is relatively low by design, as TCRs are notoriously promiscuous and capable of recognizing multiple epitopes. TCR mimics, which are antibody-like binders, have nanomolar affinities for their cognate ligand. It is possible that such high-affinity interactions may impair downstream anti-tumor activity, as recent work by the Schietinger lab suggests that high signal strength interactions lead to effector cell dysfunction [74]. Supporting these data, earlier work comparing native TCRs to TCR-mimic receptors has demonstrated that optimal function against TCR targets requires a relatively low affinity state similar to TCR–ligand interactions, and it has been demonstrated that high-affinity TCR-mimic CAR-T cells have inferior activity compared to native TCR counterparts [75,76]. It is likely that engineering strategies that tune TCR affinity based on the antigen density in normal and tumors cells will be necessary to maximize activity against each target [77]. Thus, target and therapeutic discovery cannot be performed in isolation from one another, and both must account for tumor-specific target antigen characteristics, which in turn inform optimal therapeutic design. This task requires partnership among clinicians and immunologists to better understand the mechanisms underpinning these relationships, allowing the field to further refine the preclinical vetting of candidate receptors. Antigen density on sarcoma cells will likely vary by histologic subtype, meaning that the ideal receptor avidity may as well. To summarize, consideration of the following might be helpful moving forward for clinically relevant TCR target identification in sarcomas:

1. Identifying shared or common tumor antigens derived from proteins essential for tumorigenesis.
2. Validating target presentation using HLA immunoprecipitation with mass spectrometry and consider performing these experiments invoking the immune proteasome.
3. Performing rigorous studies of both antigen density on target tumor cells as well as receptor avidity for pHLA with careful consideration of immune effector product development (i.e., TCR vs. TCR-mimic receptor).

### 2.3. Key Issues Related to Cell Product Development

The type of cell and its source dictate the utility and therapeutic efficacy. Cells can be stem or adult cells and autologous or allogeneic [78]. Although immune cells are most commonly utilized, other cell types including mesenchymal, vascular, or adipose cells can be used but are more commonly reserved for non-oncologic diseases [79]. Traditionally ACT, especially T-cell based therapies, have been autologous. However, as new strategies are developed to reduce or eliminate the restriction of HLA matching and the risk of graft-vs.-host disease, the types and sources of cell types have expanded. “Off-the-shelf” cell products offer greater generalizability with the potential for bulk manufacturing and cryopreservation, thus lowering manufacturing costs and providing an immediate option to patients. They can be sourced from healthy donors or differentiated from induced pluripotent stem cells into T cells or other immune cells [80]. Genome editing of “off the shelf” T cells, such as αβ TCR deletion, can be performed to avoid alloreactivity and other immune cells, such as allogeneic NK cells from healthy donors, can be safely used without genome editing [80,81]. Additionally, the use of allogeneic as opposed to autologous cells can ensure potent, non-exhausted healthy cells as a more optimal approach.

In early solid tumor ACT trials, T cells were most frequently used—predominantly tumor-infiltrating lymphocytes (TILs)—and later, engineered T cells such as TCR-T and CAR-T cells. While TIL therapy has been successful and is now FDA approved for use in melanoma [82], its efficacy in other solid tumors (including sarcomas) has been mixed [83,84,85]. Sarcomas are generally considered immunologically “cold” tumors due to a density of immunosuppressive myeloid cells, stromal infiltration, and a paucity of T cells that can be exhausted, dysfunctional, or effectively excluded. Attempts at and the success of TIL isolation, ex vivo culture, and expansion have been limited [86,87]. Because TIL-recognized neoantigens are patient-specific, the use of autologous cells derived from an individual’s tumor or circulation is required [81]. Despite these limitations, with the recent FDA approval of TIL therapy in melanoma, there is continued interest in expanding the application of TIL therapy to other tumors including sarcomas, and trials are ongoing.

Engineered TCR-T cell therapy has garnered recent attention with the FDA approval of afami-cel in metastatic synovial sarcoma [88]. TCR therapy can target both cell surface and intracellularly expressed proteins, providing a significant advantage over CAR-T cell therapy that targets only antigens expressed on the cell surface [35,89]. However, since TCRs require antigens to be presented by HLA specific to that epitope, patients must possess those epitope-specific HLA alleles, most commonly HLA-A*02:01, to receive these TCR-T cells [81]. Newer techniques to disrupt or replace TCR αβ chain genes can eliminate the need for HLA matching and can improve the anti-tumor activity of the therapy [80,89]. Additionally, the use of γδ T cells is now being tested as an HLA-independent option [90]. CAR-T cell therapy, in contrast, is HLA-independent but is restricted to targeting tumor surface antigens that are highly expressed. Upon activation, CAR-T cells produce a more rapid and higher level of cytokine release, resulting in a more robust anti-tumor response than TCR-T cells [91]. While potent anti-tumor activity with CAR-T cells can lead to greater treatment response, it has increased potential for significant toxicities, including cytokine release syndrome, immune effector cell hemophagocytic lymphohistiocytosis-like syndrome (IE-HS) and immune effector cell-associated neurotoxicity syndrome [91].

Additional cell types, such as NK, NKT, and myeloid cells, have been explored as HLA-independent options and offer promise. NK cells accomplish direct and indirect tumor cell killing by the release of perforins/granzymes and through antibody-dependent cellular cytotoxicity, which are independent of specific antigens and can link the adaptive and innate immune systems [81]. Unmodified autologous NK cells have been tested in multiple solid tumor trials, and while toxicities were generally manageable, efficacy has been limited, which was likely a result of their short-lived time in circulation [92,93,94]. Many investigators are now testing unmodified NK cells in combination with targeted drug therapy or CAR-modified NK cells alone or in combination to harness their highly effective killing and expansion potential married to a specific target [95]. CAR-NK cell efficacy can be greatly impacted by the balance of the activating and inhibitory receptors on the NK cell surface. Specifically, NKG2D acts as an activating receptor that is important in limiting tumor initiation and progression, and its expression can be upregulated by different cytokines in the TME [46]. Due to their limited lifespan in circulation and relative resiliency to exhaustion, CAR-NK cells have been potent and safer in clinical trials than their CAR-T cell counterparts. However, this shorter persistence affects the ability to achieve durable responses, and these NK ACT approaches will likely require repeat doses to have long-term clinical impact [46]. NKT cells are another cell type that can be modified to target tumors and possess the anti-tumor properties of both T and NK cells. While NKT cells are restricted by an HLA-like molecule, CD1d, they have not been shown to be alloreactive, which enables the use of allogenic sources. They represent <1% of circulating T cells, so methods for improved isolation and expansion are key [96]. Lastly, myeloid cells, including dendritic cells, are being explored for immunotherapy. Myeloid cells naturally traffic into and survive in tumors and can mediate an anti-tumor response through antigen presentation, the phagocytosis of dead or dying cells, the local delivery of cytotoxic substances, and the recruitment and activation of CD8^+^ T and NK cells [97]. Myeloid cells can be genetically modified to secrete anti-tumor cytokines that recruit other immune cells into the TME or target tumors through CAR activity [97,98]. As newer treatment modalities, current and upcoming clinical trials testing the safety and efficacy of myeloid cell therapies should be closely monitored.

### 2.4. Additional Opportunities in Cell Manufacturing

CAR-T cell manufacturing involves an intricate process from the extraction of T cells to genetic modification and expansion. Finding ways to optimize and streamline CAR-T cell production could help to reduce costs and increase accessibility. Despite some of the individual differences between patients, there is some heterogeneity in the phenotypes of cells used for CAR-T. By using Artificial Intelligence (AI), some of the manufacturing can be streamlined and optimized for better efficiency. Through the creation of signaling motif libraries, thousands of different CAR co-stimulatory signaling domains can be analyzed to engineer the optimal combination for each CAR and tumor type [99]. This Deep Learning (DL) can help ensure better accuracy with the identification of CAR-T cells. Zhang et al. constructed a CAR-T dataset with 500 cell images that was tested on their RCMNet classification model with a top-1 accuracy of 99.63% on a public dataset [100]. The strength of AI is that it enables the adaptive control of manufacturing to allow for a more personalized process. The field is moving toward harnessing this technology for better therapeutic outcomes. The European Union commissioned in 2020 the Artificial Intelligence-driven, Decentralized Production for Advanced Therapies in the Hospital (AIDPATH) project to develop a platform for CAR-T using these digital solutions [101]. The healthcare industry has already greatly benefited from AI both in their information systems as well as financially. The feasibility of implementing a rapid manufacturing process for CAR-T in clinical settings could be transformative, but the cost of operating the AI infrastructure is important for hospital systems to consider. Additionally, there needs to be precise regulation to ensure the validity and reliability of these AI algorithms.

**Figure 2:**
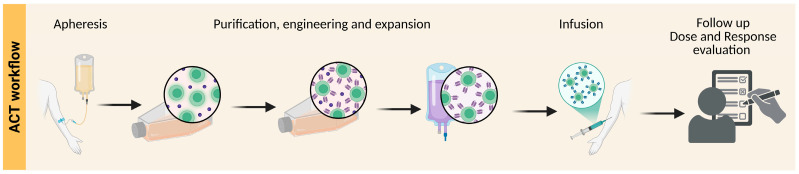
Cell product production begins with blood draw, often in the form of a apheresis (left); a certain cell type may be purified, engineered and expanded before ultimately being re-infused back to the patient. Created in BioRender by Oliva-Ramírez, J. (2025) www.biorender.com (accessed on 1 September 2025).

Since the first FDA approval of T cell-based therapy, the field of cellular immunotherapy has greatly expanded and now includes many more cell types and approaches. While two cellular therapies have recently been approved for solid tumor indications (one for synovial sarcoma) [82,88], continued investigations and new strategies are critically needed to augment the clinical efficacy of ACT in solid tumors. These strategies include improvement in cell manufacturing techniques, conditioning, and combinatorial approaches with cytokines, agents targeting the immunosuppressive TME, or an additional cellular therapy (Figure 2).

### 2.5. Conditioning Regimens

Conditioning chemotherapy regimens play an important role in delivering cell therapies. These regimens vary greatly across studies and institutions and are reviewed elsewhere [102]. The primary role of conditioning chemotherapy is to deplete regulatory lymphocytes and stimulate cytokine production to support the engraftment of the transferred cells. A second role is the cytoreductive effects, although their relevance to subsequent immunotherapy responses is less clear. Some sarcomas are highly sensitive to alkylating agents, such as cyclophosphamide, which is frequently used during conditioning and can have significant anti-tumor activity. This ‘debulking’ of tumors may improve the effectiveness of cell therapy but is a confounding variable for evaluating the efficacy of cell therapies. As an example, the extent of response for synovial sarcoma and myxoid round cell liposarcoma patients to MAGE-A4 TCR-T cells (afami-cel) is confounded by potential lingering cytoreductive effects of the conditioning chemotherapy [103]. Evidence of such an effect was demonstrated in an anti-CD19 CAR-T cell trial in which the debulking of patients with intensive chemotherapy prior to lymphodepletion significantly improved overall survival in patients with relapsed/refractory diffuse large B-cell lymphoma [104]. Overall, the sarcoma field would benefit from standardizing the conditioning regimens used in the setting of T cell therapies.

### 2.6. Timing of Cell Product Administration

Tumor burden is perhaps the most broadly shared correlate with responsiveness to cell therapy. While there are limited data for sarcomas, cell therapy for solid tumors and hematological malignancies have supported a low-disease burden state as a positive predictor of response. Results from the GD2-CART01 cell therapy trial for neuroblastoma indicated that patients with high disease burden had no responses compared to 67% of low disease burden patients at 3 years [105]. In a cohort of 78 B-ALL patients treated with CD19 CAR-T cell therapy, high tumor burden was associated with a 16% lower CR rate [106]. A larger study of 185 B-ALL patients treated with tisagenlecleucel had a similar finding with a 1-year OS of 85% in low disease burden patients compared to 58% in high disease burden patients [107]. A similar association was found in synovial sarcoma and myxoid round-cell liposarcoma patients treated with MAGE-A4 TCR-T cell therapy [38].

These findings support moving cell therapy upfront for high-risk sarcomas once a minimal residual disease state is attained. Some sarcomas, particularly among the pediatric and adolescent/young adult population, are highly responsive to chemotherapy and can undergo complete response but have a high relapse rate. It is conceivable that adoptive T cell therapy may be most effective at this stage of treatment. A tradeoff of this approach which needs to be considered is that the lack of evaluable disease sites makes interpreting clinical responses challenging. Comparisons to historical outcome data are challenging since treatments continue to evolve, and the general improvement in providing care for cancer patients can lead to an overestimation of the effectiveness of cell therapy. Considering the financial cost of cell therapy, having clear evaluable disease sites will continue to be important for testing new cell therapies. It also remains unclear, particularly for sarcomas, whether high tumor burden alters the functionality of infused T cells or if it simply reflects a more aggressive disease state. Correlative studies in future cell therapy trials may help distinguish efficacy from tumor burden, the T cell therapy phenotype, and sarcomas’ intrinsic properties.

### 2.7. Cell Therapy in Patients Requiring Other Targeted Therapy

The use of receptor tyrosine kinase inhibitors routinely used for the treatment of GIST and many other sarcomas may also play a potential role in potentiating cellular therapy. In animal models, imatinib depleted CD4^+^ regulatory T cells which improved immunotherapy response [108]. MHC-I loss in GIST was frequently observed in patients after imatinib treatment, which may represent tumor editing by an active immune response [109]. Moreover, imatinib therapy was associated with a decrease in IDO, which improved CD8^+^ T cell activation in resected GIST [110]. As such, immune activation may be an important component of the therapeutic efficacy of imatinib. This raises important questions, however, about highly effective therapy against driver oncogenes and any associated favorable immune responses. Further studies are clearly needed to understand the immunomodulatory effect of kinase inhibitors given prior to or concurrently with T cell therapy.

### 2.8. Persistence and Expansion of Cell Therapies in Patients After Administration

Adoptively transferred T cell persistence and expansion is of utmost importance to understand, as both factors are associated with treatment outcomes for sarcoma patients. In clinical trials of patients with synovial sarcoma treated with afami-cel, most patients exhibited a peak around the first week, but there was a considerable variation in persistence as measured by copies per µg of DNA after infusion [38]. Interestingly, overall survival was prolonged with greater T cell persistence based on transferred TCR gene expression over the initial 3 months after the infusion [38]. Similar results were seen in patients with sarcoma receiving HER2-specific CAR-T cells with higher levels at week 1 determined by transgene detection [111]. Few studies report intra-tumoral CAR-T cells and their relation to the quantity or functional state in peripheral blood. Several studies in non-sarcoma tumors have observed CAR-T cell trafficking to tumors in lower concentrations compared with peripheral blood [112,113,114]. The identification of strategies to improve T cell trafficking into the TME is an important goal for the field.

The analysis of peripheral CAR-T cells in adjacent sites, such as ascites or cerebrospinal fluid, has also been explored as part of biodistribution. Hass et al. reported 29 days of persistence in two of five patients, and DNA copies were detected in peritoneal fluid on day 14, with higher copies than in blood [115]. Similarly, GD2-CART01 cells persisted in cerebrospinal fluid 12 weeks after infusion in five patients and persisted over 2 years in bone marrow [105]. Nevertheless, the relationship of CART levels in different tissue sites to clinical outcome is unclear due to the small number of patients analyzed. Further evaluation and standardization are necessary, including the ratios between blood and tissue and other biodistribution, which will allow us to better understand CAR-T trafficking and correlate the findings to clinical benefit.

Instead of tracking bulk numbers of CAR-T cells, some studies have assessed populations of different T cell subsets like effector or central memory in the infused product or circulation by assaying CD45RA, CD45RO, CCR7, and CD27 staining and relating their expansion to clinical benefit [112,116,117]. The potential of T cell subpopulations as biomarkers is intriguing and requires further research. Products with lower CD45RA^+^CCR7^−^ (T effector memory) frequency were more likely to achieve longer PFS in one analysis [116]. In the recent report of GD2-CART01, patients with central and effector memory CAR-positive CD4 and CD8 cells had prolonged persistence [105]. Hegde et al. demonstrated that patients with advanced sarcoma that demonstrated complete response to HER2-CAR T cells had CD45RA^+^CD27^−^ cells. Moreover, infusion products with higher amounts of inhibitory markers like LAG3, TIM-3, or CD39 were associated with progressive or stable disease [111]. Exploring specific populations in the CAR-T product before and after infusion could reflect the overall expansion of memory and effector subsets rather than naive populations, which might represent a biomarker of response.

### 2.9. Tumor Microenvironment

The sarcoma TME is a complex, dynamic, and hostile “ecosystem” whose cellular and non-cellular components pose several barriers to CAR-T cell trafficking and effectiveness [118]. Tumor cells themselves directly interfere with CAR-T cell function via the expression of inhibitory immune checkpoint signals, such as PD-L1 and PD-L2 [119,120,121]. Tumor cells can influence or take advantage of nearly all components of the TME (including immune cells, stromal components, metabolic changes, and the tumor vasculature) to hinder CAR-T cell efficacy.

To successfully traffic to the tumor, peripherally infused CAR-T cells must first traverse the tumor vasculature. This process requires the production of appropriate chemokines by the endothelial cells and tumor stroma, namely CXCL9, CXCL10, CXCL11, and CCL5 [122]. Next, T cells must transmigrate through the endothelial cells to penetrate into the tumor stroma, again under the direction of chemokine gradients including CXCR3 and CCR5. Once within the parenchyma, T cells must navigate a dense extracellular matrix (ECM), fibroblast and myeloid-mediated immunosuppression (e.g., via TGF-β), and aberrant vasculature to reach tumor cells, recognize them, and exert their cytotoxic effects [123,124,125].

Even after successfully infiltrating the tumor, CAR-T cells must contend with potent immunosuppressive forces within the TME. The most well-described cellular barriers to CAR-T cells in the TME are immune cells, particularly immunosuppressive myeloid cells and regulatory T cells. Tumor-associated macrophages (TAMs) comprise one type of immunosuppressive myeloid cell in the sarcoma TME [122,123]. TAMs function on a spectrum that spans between two major differentiation classes: M1-like macrophages that are typically pro-inflammatory and exhibit anti-tumor functions and M2-like macrophages that suppress anti-tumor immune cells and support tumor growth through the production of pro-angiogenic and pro-tumorigenic growth factors [124,125]. Importantly, TAM differentiation is dynamic, and macrophages can be therapeutically reprogrammed [126]. MDSCs and regulatory T cells suppress CAR-T cell activity primarily through the secretion of inhibitory cytokines and chemokines, such as arginase 1 (ARG1), transforming growth factor (TGF)-β, interleukin (IL)-10, and indoleamine 2,3-dioxygenase (IDO). Moreover, myeloid cells can directly inhibit CAR-T cells by expressing immune checkpoint ligands [127,128,129]. Lastly, in addition to immune-mediated suppression, metabolic barriers in the TME such as hypoxia, acidosis, and nutrient depletion also impair CAR-T cell function. Tumor-driven competition for glucose, glutamine, and other amino acids, along with the production of immunosuppressive metabolites like lactate and kynurenine, hinders CAR-T cell proliferation and persistence [130,131]. Together, these results demonstrate that there are many challenges but also great opportunities in targeting the TME to enable more effective cellular therapy. Future efforts using spatial transcriptomics combined with cutting edge AI-powered analytics may lead to the discovery of key biomarkers in the TME.

### 2.10. Biomarkers for CAR-T Therapies in Solid Tumors

RECIST imaging is broadly used in sarcoma clinical trials, but many investigators feel it is inadequate, and developing improved measurements to evaluate CAR-T cell efficacy is another opportunity for translational research. The challenges are the lack of a consensus on time points, sample types, analytes, and technologies, all of which are vital for discovering biomarkers. However, the potential of new biomarkers offers hope and optimism for the future of CAR-T therapies. What we have learned from CAR-T therapy success in blood malignancies by tracking clinical response cannot be fully reproduced for solid tumors, considering their different biology. For example, in blood malignancies with durable responses and high levels of persistent cells in the blood, the peak of peripheral blood CAR-T cell expansion is around 7–14 days [132]. The difference in circulating CAR-T for solid tumors is generally around five to ten-fold lower with a variable expansion peak, supporting the need for better in situ biomarkers related to clinical responses as summarized by Albelda [133]. High CAR-T in the circulation is desirable when cancer arises from blood cell precursors. However, this is not the scenario for solid tumors, where cell persistence and expansion, in both the tumor and draining lymph nodes, are the goals. We can divide the approaches to biomarker investigations into categories based on the nature and exploratory level available from clinical trials in solid tumors.

Lower tumor burden defined by clinical and radiographic assessment positively impacts response prediction. In the clinical trial phase1/2 with GD2-CART01 for neuroblastoma, lower disease burden at infusion predicted response and survival compared to those with higher burden [105]. In synovial sarcoma, responders mainly comprised females with higher MAGE-A4 antigen expression and lower disease burden before lymphodepletion [111]. Also, tumor burden has been explored as a risk factor for cytokine release syndrome (CRS) in blood and solid malignancies [38,105,111,134]. Markers such as PD-L1 and tumor mutation burden (TMB) have been proposed in the context of cellular therapy, as these may indicate a more inflammatory phenotype in the tumor, though even in the context of immune checkpoint blockade therapy, their use can be controversial [135,136].

The identification of blood-based biomarkers to predict the success of cellular therapy is another key goal for the field. For instance, Hassan et al. found in patients with mesothelin-expressing solid tumors that serum levels of soluble mesothelin-related peptides (SMRPs) and megakaryocytic potentiating factor (MPF) decreased with treatment and were significantly lower in patients with radiographical response [137] and similar findings have been seen in prostate cancer patients and those with brain tumors [112,114]. Other studies have found myeloid markers associated with response in GD2 CART patients with immunosuppressive myeloid cells associated with poor CAR expansion and CXCR3 myeloid cells associated with good expansion [138]. Some investigators have examined tracking CAR-T cells designed to recognize soluble antigens such as TGFβ. In preclinical or clinical studies, TGFβ reduction was related to an indirect CAR-T cell effector activity [139]. Soluble MUC16 or HER2 have been used for tracking specific CAR-T effects [111,140]. Circulating tumor DNA (ctDNA) has been shown to correlate with disease burden and identify responders to immune checkpoint inhibitors in sarcomas and other solid tumors [141]. Furthermore, several studies have also shown that ctDNA analysis can identify patients responding to CAR-T cell therapy and potentially identify mechanisms of treatment resistance [142,143,144]. Ultimately, these biomarkers hold promise for identifying patients most likely to benefit from ACTs to maximize efficacy and minimize toxicity.

## 3. Conclusions

In many ways, sarcomas are an ideal disease target for the development of ACT as demonstrated by the great success of afami-cel in synovial sarcoma patients. However, there is a great deal of work to be completed in order to improve ACT and bring its benefits to all sarcoma patients. The identification of novel targets is a critical issue as well as identification of the ideal cell types for use in therapy. Future efforts to make manufacturing more efficient and cost effective may help overcome the logistic barriers which remain significant for these therapies. Better conditioning and combination post-treatment therapies including TKIs may yet further improve ACT efficacy, and a deeper understanding of the TME may help us best design these strategies. Biomarkers may help us treat patients most appropriately. Despite considerable challenges, we are optimistic that ACT will improve and deliver deeper and durable responses to more sarcoma patients.

## Abbreviations

The following abbreviations are used in this manuscript:

ACTs	Adoptive Cellular Therapies
SASS	Strategic Advances in Sarcoma Science
TME	Tumor Microenvironment
CARs	Chimeric Antigen Receptors
MHC	Major Histocompatibility Complex
TCRs	T Cell Receptors
PC	Peptide-centric
Lete-cel	Letetresgene autoleucel
IL1RAP	Interleukin-1 Receptor Accessory Protein
NK	Natural Killer
MDSCs	Myeloid-Derived Suppressor Cells
TAAs	Tumor-Associated Antigens
TDAs	Tumor Differentiation Antigens
TSAs	Tumor-Specific Antigens
pHLA	Peptide-Human Leukocyte Antigen
NGS	Next-Generation Sequencing
AML	Acute Myeloid Leukemia
TILs	Tumor-Infiltrating Lymphocytes
AI	Artificial Intelligence
DL	Deep Learning
AIDPATH	Artificial Intelligence-driven, Decentralized Production for Advanced Therapies in the Hospital
ECM	Extracellular Matrix
TAMs	Tumor-Associated Macrophages
ARG1	Arginase 1
TGF	Transforming Growth Factor (TGF)-β
IL	Interleukin-10
IDO	Indoleamine 2,3-dioxygenase
TMB	Tumor Mutation Burden
SMRP	Soluble Mesothelin-Related Peptides
MPF	Megakaryocytic Potentiating Factor
ctDNA	Circulating Tumor DNA
